# Comparing the Prognostic Utility of Vertebral and Endplate Bone Quality Scores for Cage Subsidence Following Single‐Level Anterior Cervical Discectomy and Fusion: A Retrospective Analysis

**DOI:** 10.1111/os.70260

**Published:** 2026-02-12

**Authors:** Omar Lubbad, Wajeeh Ullah Mahmood, Rafiq Sheikhali, Mohammad Abir Mamun, Akram Hagos, Laila Lubbad, Suzanne Murphy, Giuseppe Lambros Morassi, Nektarios K. Mazarakis

**Affiliations:** ^1^ Brighton and Sussex Medical School Brighton UK; ^2^ Royal College of Surgeons in Ireland Medical School Busaiteen Bahrain; ^3^ Department of Surgery Royal College of Surgeons Ireland Dublin Ireland; ^4^ Department of Neurosurgery Beaumont Hospital Dublin Ireland; ^5^ Department of Neurosurgery Royal Sussex County Hospital Brighton UK; ^6^ Royal College of Surgeons in Ireland Medical School Dublin Ireland

**Keywords:** anterior cervical discectomy and fusion, bone quality, cage subsidence, magnetic resonance imaging

## Abstract

**Objective:**

Cage subsidence following anterior cervical discectomy and fusion (ACDF) is linked with poor bone quality. MRI‐derived bone quality scores have been shown to provide valuable insights into postoperative complication risk; however, the optimal MRI‐based metric for predicting cage subsidence remains unclear. This study aims to compare the predictive value of different MRI‐derived bone quality measures for cage subsidence following ACDF.

**Methods:**

Patients undergoing single‐level ACDF between October 2012 and September 2022 at our institution with at least 6 months of radiographic follow‐up were retrospectively evaluated. T1 preoperative MRI scans were used to measure mean, median, and segmental vertebral bone quality (VBQ) scores, and upper, lower, and average endplate bone quality (EBQ) scores. Postoperative and follow‐up X‐rays were used to identify cage subsidence.

**Results:**

Fifty‐six patients met the inclusion criteria; 26 developed cage subsidence and 30 did not. Age, sex, surgical indication, cage type, and clinical setting were similar between groups. Mean disc space loss was significantly greater in the subsidence group (3.99 mm vs. 0.37 mm; *p* < 0.001). All bone quality scores were significantly higher in the subsidence group across all metrics. Mean VBQ (OR = 14.22), segmental VBQ (OR = 8.23), and lower EBQ (OR = 5.54) were strong predictors of subsidence (*p* < 0.001). ROC analysis showed excellent discrimination for mean VBQ (AUC = 0.821), segmental VBQ (AUC = 0.817), and median VBQ (AUC = 0.817). Interobserver reliability was high for all bone quality metrics (ICC 0.836–0.925).

**Conclusion:**

MRI‐derived bone quality metrics, particularly VBQ and lower endplate EBQ scores, are strong predictors of cage subsidence following single‐level ACDF. These findings reinforce the clinical utility of preoperative MRI as a non‐invasive, radiation‐free tool for assessing vertebral bone integrity. Incorporating VBQ and EBQ assessments into surgical planning may enhance risk stratification and optimize postoperative outcomes in patients undergoing cervical fusion.

## Graphical abstract

MRI‐derived bone quality metrics are reliable predictors of cage subsidence following ACDF. Their implementation may be used to guide surgical planning and improve outcomes.

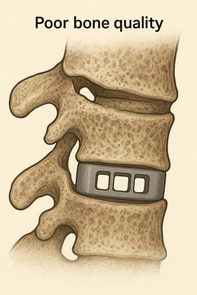

## 1 Introduction

Cage subsidence, the migration of an interbody cage into the vertebral bodies, is the most common hardware‐related complication of anterior cervical discectomy and fusion (ACDF) and spinal fusion surgeries in general [1]. Such significant loss of intervertebral disc space height can have substantial effects on the biomechanics of the cervical spine, potentially manifesting with loss of lordosis at the cervical, segmental, and proximal joint levels [1, 2]. Such kyphotic changes and sagittal malalignment may also accelerate degenerative changes at adjacent levels, necessitating further operations [2]. Moreover, cage subsidence has been associated with an increased risk of pseudarthrosis, underscoring its significance as an indicator of poorer fusion outcomes and overall prognosis [3]. While the incidence of subsidence is considered multifactorial, poor bone quality has repeatedly been suggested as a risk factor [4, 5].

Despite dual‐energy X‐ray absorptiometry (DEXA) remaining the gold standard for assessing bone mineral density, it is not commonly performed for patients awaiting spinal fusion due to feasibility, cost‐effectiveness, and the benefits of doing so prior to spine surgery have not yet been established [6]. Additionally, access to DEXA scans may be limited or considered impractical across some healthcare systems. For such reasons, alternative methods for assessing bone quality have recently emerged, offering comparable accuracy and convenience. Of these, a magnetic resonance imaging (MRI)‐derived vertebral bone quality (VBQ) score specific to the spine has demonstrated significant clinical utility [7]. For example, VBQ scores have shown strong diagnostic accuracy for discriminating between patients with and without osteoporosis [7]. This score can be derived from T1‐weighted MRI scans, which are routinely performed before ACDF, offering valuable insight without any additional cost or radiation exposure. Higher VBQ scores suggest increased fatty infiltration into vertebral bodies, indirectly reflecting poor bone quality and mineral density [8].

The predictive value of VBQ scores for cage subsidence has been well‐established throughout the literature, with various studies proving its utility across multiple types of spinal fusion. Despite this, several methodologies have been described for calculating VBQ scores, varying in terms of region‐of‐interest placement, vertebral levels measured, and calculation methods. A similar MRI‐derived endplate bone quality (EBQ) score has also been linked to cage subsidence following spinal fusion, reflecting bone quality directly from the endplates in contact with the cage [9]. While several studies have supported its predictive value, the literature remains inconclusive regarding whether any specific methodology confers a significant advantage in accuracy, reliability, or clinical applicability for predicting cage subsidence. Therefore, the purposes of this study were to (i) compare the predictive performance of vertebral and endplate MRI‐derived bone quality metrics for cage subsidence following single‐level anterior cervical discectomy and fusion, (ii) evaluate whether global versus segmental vertebral bone quality measurements differ in their discriminative accuracy and strength of association, and (iii) assess the interobserver reliability of these techniques to identify the most reproducible and clinically applicable method.

## 2 Methods

### 2.1 Study Design

This retrospective cohort study was conducted at a single center and received approval from the Royal Sussex County Hospital internal clinical governance committee (project ID: 2581). Patients who underwent anterior cervical discectomy and fusion (ACDF) between October 2012 and September 2022 were evaluated for eligibility. Inclusion criteria were: (1) age over 18 years, (2) availability of preoperative MRI performed within 1 year before surgery, (3) postoperative cervical radiograph obtained within 1 week of surgery, and (4) follow‐up cervical radiographs taken at least 6 months postoperatively. Patients were excluded if they had undergone multilevel fusion, had a history of previous cervical spine surgery, or lacked adequate imaging.

### 2.2 Surgical Technique

All procedures were performed by the same group of surgeons at our institution. Interbody fusion was achieved using either polyetheretherketone (PEEK) or titanium cages, both filled with autologous bone graft. The use of anterior cervical plating was documented. All patients received standard postoperative care, including routine mobilization and follow‐up.

### 2.3 Score Measurements

**FIGURE 1:**
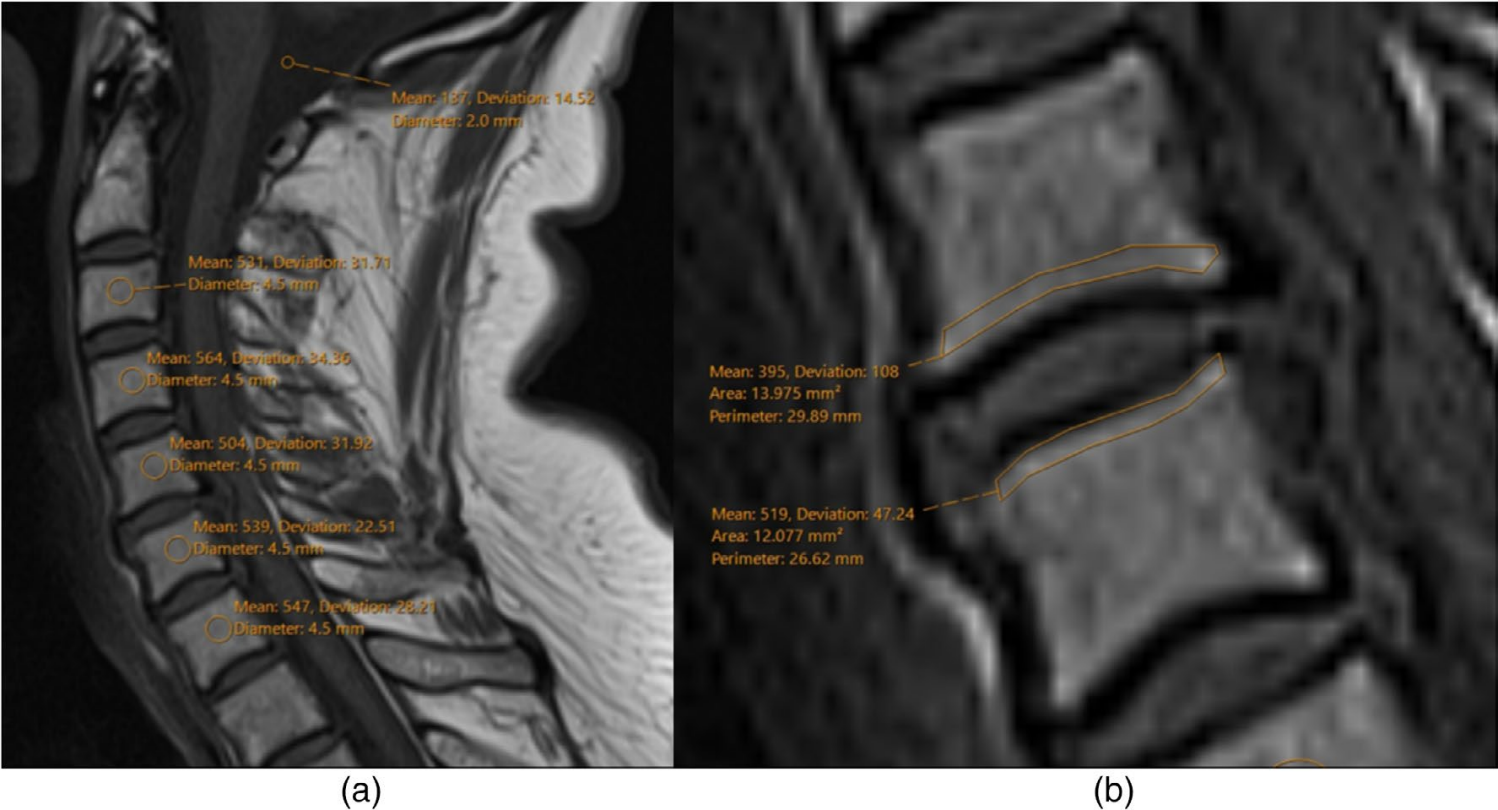
VBQ measured across C3–7 with CSF standardisation at the Cisterna Magna (a) and EBQ score measurement at operated levels (b) from preoperative mid‐sagittal MRI.

Preoperative midsagittal T1‐weighted MRI scans were used to calculate VBQ and EBQ scores. Signal intensities (SI) were measured using 4.5 mm regions of interest (ROI) placed within the cancellous bone of the C3–C7 vertebral bodies, carefully avoiding the endplates and cortical margins for VBQ scores [10]. Endplate SIs were measured by manually outlining the superior and inferior endplates, avoiding cortical margins, degenerative changes, and Schmorl's nodes [11]. Cerebrospinal fluid (CSF) SI was measured using two 2 mm ROIs placed in the cisterna magna. Global mean and median VBQ scores were derived from the vertebral SIs C3–C7, divided by the average CSF SI. Segmental VBQ scores were measured from an average of the SIs of the operated levels. Lower and upper EBQ scores were measured by using the SIs of upper and lower endplates; EBQ scores were measured by using an average of both. Measurements were performed independently by two observers. In cases where the difference between observers exceeded 10%, the scan was jointly reviewed, and a consensus was reached (Figure 1).

### 2.4 Radiological Outcomes

**FIGURE 2:**
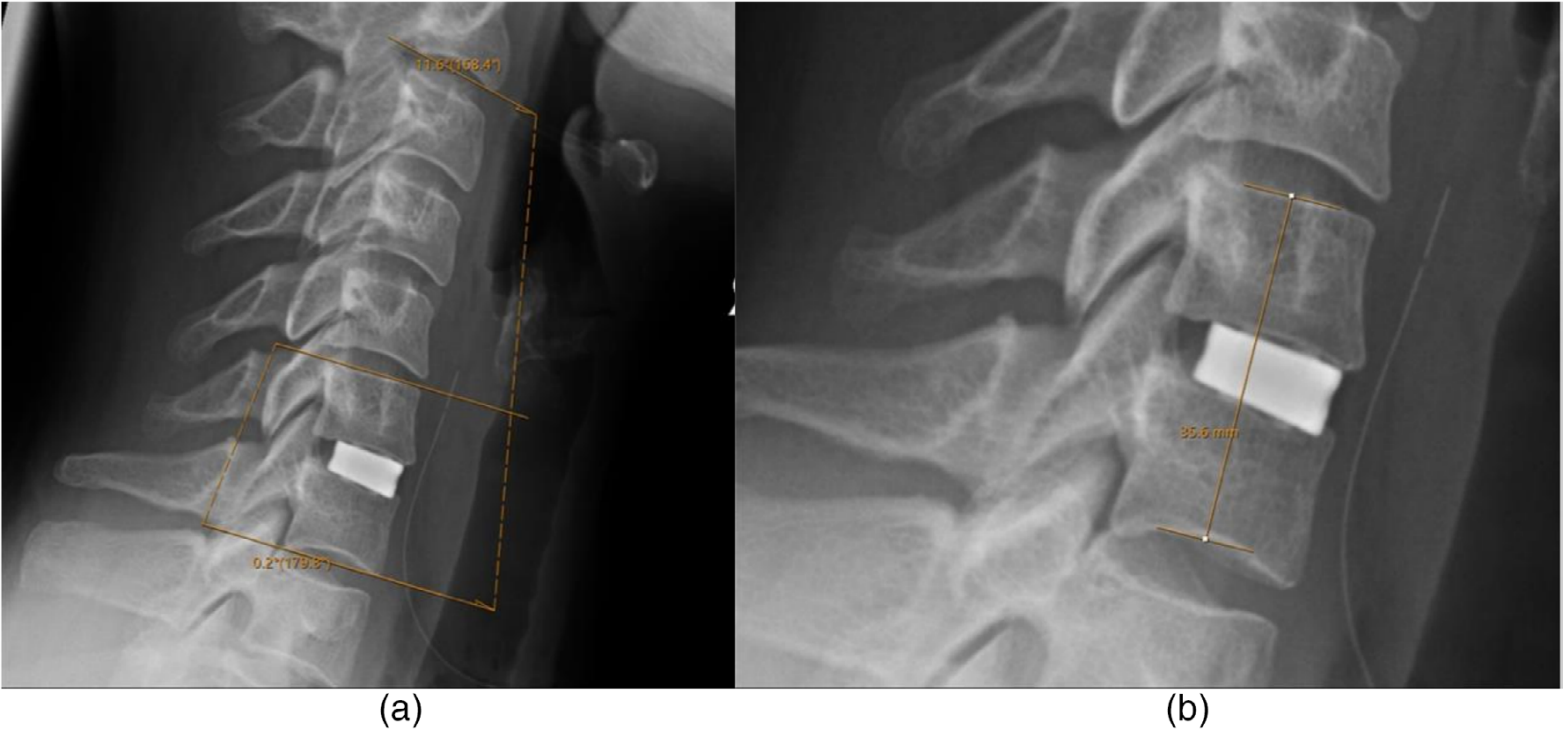
Cervical C2–7 and segmental cobb angle measurements (a) and segmental height measurement across the fused level (b) from neutral lateral radiographs.

Cage subsidence was defined as a decrease of 2 mm or more in intervertebral height from the immediate postoperative scan to follow‐up imaging [10]. We selected a 2 mm threshold given our minimum follow‐up of 6 months to improve sensitivity for detecting early subsidence changes in this patient cohort. Measurements were taken from the superior endplate of the upper fused vertebra to the inferior endplate of the lower fused vertebra. Cobb angles were calculated between the inferior endplates of C2 and C7, as well as across the fused segments and their proximal adjacent levels, to assess cervical, segmental, and proximal sagittal alignment [12]. Loss of lordosis was defined as a reduction of 5° or more in the respective Cobb angles over the follow‐up period (Figure 2).

### 2.5 Statistical Analysis

Statistical analyses were performed using SPSS version 29.0 (IBM Corp., NY, USA). Continuous variables were reported as mean ± standard deviation (SD) or median with interquartile range (IQR), while categorical variables were presented as frequencies and percentages. The Shapiro Wilks test was used to assess for normal distribution. The independent *t*‐test or Mann–Whitney U tests were used to compare VBQ scores between patients with and without cage subsidence or loss of lordosis. Categorical data were analyzed using the chi‐square test or Fisher's exact test as appropriate. Multivariate logistic regression was employed to determine whether VBQ scores were an independent predictor of cage subsidence, adjusting for age, sex, cage type, and surgical urgency (elective vs. emergency). Receiver operating characteristic (ROC) curve analysis was performed to evaluate the diagnostic accuracy of VBQ scores, with area under the curve (AUC) values reported. A *p*‐value < 0.05 was considered statistically significant. Interobserver reliability was assessed using the intraclass correlation coefficient (ICC), based on a two‐way mixed‐effects model with absolute agreement.

To compare the discriminatory performance, DeLong's test for two correlated ROC curves was conducted in R version 4.4.1 (R Foundation for Statistical Computing, Vienna, Austria) using the pROC package (roc.test() with method = “delong”). Pairwise comparisons of AUCs were adjusted for multiple testing using the Bonferroni correction. Additionally, each continuous variable was dichotomized at its optimal cutoff value derived from Youden's index. These binary classifications were then compared pairwise using McNemar's test (mcnemar.test() in R) to assess differences in discordant classification outcomes between predictors. A *p*‐value < 0.05 was considered statistically significant.

## 3 Results

### 3.1 Patient Characteristics

A total of 56 patients who underwent single‐level ACDF were included in the study, with 26 developing cage subsidence and 30 showing no subsidence. The average age was slightly higher in the subsidence group (59.0 years) compared to those without subsidence (57.1 years), but this difference was not statistically significant. The gender distribution was similar between groups (*p* = 0.643). Patients in the subsidence group showed a higher prevalence of diabetes and a trend toward increased hypertension, although neither reached statistical significance (*p* = 0.06). Recent steroid use did not differ meaningfully between the two groups (Table 1).

**TABLE 1:** Patient demographics.

Demographic data	Subsidence (*n* = 26)	No subsidence (*n* = 30)	Statistic (*t*/*χ* ^2^)	*p*
Age	59.02 (11.86)	57.059 (13.51)	0.573	0.569
Female: Male	12:14	12:18	0.22	0.643
Diabetes	7	2		0.06
Hypertension	9	4	3.54	0.06
Steroid use < 1 year of operation	6	5	0.36	0.547
Smoking	11	11	0.19	0.66
Indication				
Canal stenosis/myelopathy	12	9	5.95	0.311
Disc prolapse/herniation	7	15		
Discitis	1	0		
Foraminal stenosis	4	2		
Trauma/fracture	2	3		
Other	0	1		
Clinical setting				
Elective	20	23	0.001	0.982
Emergency	6	7		
Operated levels				
C3/4	10	8	4.04	0.4
C4/5	2	5		
C5/6	11	9		
C6/7	3	7		
C7/T1	0	1		
Cage material				
PEEK cage	17	23	0.87	0.351
Titanium cage	9	7		
Implant configuration				
Standalone	19	22	0.58	0.748
Zero‐profile	4	3		
Plate	3	5		
Imaging				
Average time between MRI and operation (months)	2.07 (3.1)	2.03 (2.6)	0.54	0.956
Average FU scan time (y)	2.66 (2.54)	2.54 (2.61)	0.17	0.864
Average disc space loss (mm)	3.99 (1.91)	0.37 (1.32)	9.1	< 0.001
Loss of cervical lordosis	10/25	5/26	2.65	0.104
Loss of segmental lordosis	11/26	6/28	2.73	0.099
Loss of proximal junctional lordosis	2/16	2/20	0.056	0.813
Global mean VBQ	2.798 (0.4178)	2.148 (0.533)	4.91	< 0.001
Segmental VBQ	2.7982 (0.491)	2.122 (0.618)	4.48	< 0.001
Global median VBQ	2.8003 (0.444)	2.151 (0.552)	4.79	< 0.001
EBQ	2.08 (0.514)	1.745 (0.511)	2.44	0.009
Upper EBQ	2.504 (0.673)	2.057 (0.632)	2.56	0.007
Lower EBQ	2.620 (0.664)	1.967 (0.566)	3.98	< 0.001

Surgical variables were largely comparable. There were no significant differences in surgical indication, urgency (elective vs. emergency), cage type, implant configuration, or the levels fused. The interval between the last preoperative MRI and follow‐up imaging was also similar. While patients with subsidence appeared to have greater loss of cervical and segmental lordosis, this difference was not statistically significant (Table 1).

### 3.2 Bone Quality Scores and Subsidence

Patients who developed cage subsidence had significantly higher bone quality scores across all measured metrics. The mean global VBQ score in the subsidence group was 2.798 ± 0.4178, compared to 2.148 ± 0.533 in the non‐subsidence group (*p* < 0.001). Similarly, segmental (2.798 ± 0.491 vs. 2.122 ± 0.618; *p* < 0.001) and median (2.8 ± 0.444 vs. 2.151 ± 0.552; *p* < 0.001) VBQ were elevated in the subsidence group. EBQ scores also differed significantly, with higher values observed in patients with subsidence (2.08 ± 0.514 vs. 1.745 ± 0.511; *p* = 0.009). Analysis of upper and lower endplate scores showed comparable trends, with upper EBQ scores of 2.504 ± 0.673 versus 2.057 ± 0.632 (*p* = 0.007), and lower EBQ scores of 2.62 ± 0.664 versus 1.967 ± 0.566 (*p* < 0.001) in the subsidence and non‐subsidence groups, respectively (Figures 1 and 2).

### 3.3 Logistic Regression

In univariate logistic regression, all bone quality metrics were significantly associated with cage subsidence. The strongest predictors were mean VBQ (OR = 14.22; 95% CI: 3.17–63.93; *p* < 0.001), median VBQ (OR = 14.63; 95% CI: 3.26–65.76; *p* < 0.001), and segmental VBQ (OR = 8.23; 95% CI: 2.46–27.51; *p* < 0.001). As for the EBQ scores, LEBQ demonstrated the strongest association (OR = 5.54; 95% CI: 1.96–15.65; *p* = 0.001). In contrast, differences in patient and operative characteristics such as age, sex, smoking, cage type, plating, and clinical setting were not statistically significant predictors of subsidence in univariate and multivariate models (Table 2).

**TABLE 2:** Logistic regression for subsidence.

Predictor	OR	95% CI	*p*	Multivariate OR	95% CI	*p*
Age	1.013	0.971–1.056	0.562	1.02	0.975–1.068	0.385
Sex	1.286	0.445–3.719	0.643	1.248	0.425–3.668	0.687
Cage material	1.739	0.540–5.604	0.354	2.062	0.593–7.167	0.255
Smoking	1.267	0.432–3.713	0.667			
Clinical setting	1.014	0.292–3.521	0.982			
Plating	0.909	0.217–3.815	0.896			
Zero profile cage	1.544	0.306–7.785	0.599			
VBQ	14.223	3.165–63.926	< 0.001			
UEBQ	2.952	1.186–7.347	0.020			
LEBQ	5.542	1.963–15.652	0.001			
EBQ	3.675	1.177–11.480	0.025			
MEDVBQ	14.631	3.255–65.760	< 0.001			
SEGVBQ	8.229	2.462–27.509	< 0.001			

### 3.4 ROC Analysis

**FIGURE 3:**
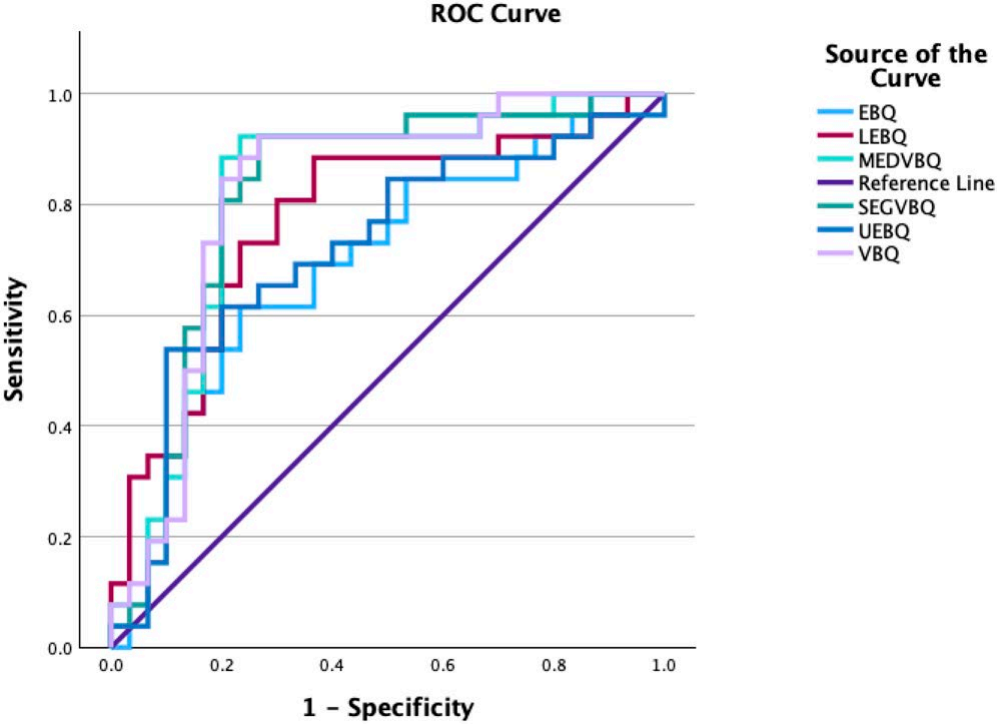
Combined ROC curve for all metrics demonstrating superior discrimination for VBQ based metrics.

ROC curve analysis showed excellent discriminative performance for mean VBQ (AUC = 0.821; 95% CI: 0.702–0.939), median VBQ (AUC = 0.817), and segmental VBQ (AUC = 0.817), all with *p* < 0.001. These scores achieved high sensitivity (0.923) with acceptable specificity (0.733–0.767). Lower EBQ also had a good AUC (0.773; 95% CI: 0.646–0.900), while UEBQ had strong specificity (0.900) but lower sensitivity (0.538). EBQ showed the weakest performance with an AUC of 0.696 and a specificity of only 0.615 (Table 3, Figure 3).

**TABLE 3:** ROC analysis results.

Variable	AUC	95% CI	Cutoff	Sensitivity	Specificity	*p*
Mean VBQ	0.821	0.702–0.939	2.365	0.923	0.733	< 0.001
Median VBQ	0.817	0.697–0.937	2.428	0.923	0.767	< 0.001
Segmental VBQ	0.817	0.697–0.937	2.329	0.923	0.733	< 0.001
EBQ	0.696	0.557–0.836	1.9676	0.615	0.767	0.006
UEBQ	0.718	0.578–0.857	2.568	0.538	0.9	0.002
LEBQ	0.773	0.646–0.9	1.922	0.885	0.633	< 0.001

### 3.5 Pairwise Score Comparison

Pairwise DeLong tests demonstrated that mean and median VBQ scores significantly outperformed EBQ and UEBQ in discriminative ability based on raw *p*‐values (e.g., mean VBQ vs. UEBQ: *p* = 0.0425; mean VBQ vs. EBQ: *p* = 0.0485). However, after Bonferroni correction, none of the comparisons remained statistically significant (adjusted *p*‐values > 0.6 across all pairs), indicating no definitive difference in AUC performance between predictors (Table 4).

**TABLE 4:** Delong's test results.

Predictor1	Predictor2	AUC1	AUC2	Raw *p*‐value	Adjusted *p* (Bonferroni)
MEANVBQ	EBQ	0.8192	0.6955	0.0485	0.728
MEANVBQ	LEBQ	0.8192	0.7731	0.3536	1.0
MEANVBQ	UEBQ	0.8192	0.7173	0.0425	0.6373
MEANVBQ	SEGVBQ	0.8192	0.8167	0.8671	1.0
MEANVBQ	MEDVBQ	0.8192	0.8167	0.8034	1.0
EBQ	LEBQ	0.6955	0.7731	0.1785	1.0
EBQ	UEBQ	0.6955	0.7173	0.6957	1.0
EBQ	SEGVBQ	0.6955	0.8167	0.0464	0.6954
EBQ	MEDVBQ	0.6955	0.8167	0.0503	0.7542
LEBQ	UEBQ	0.7731	0.7173	0.1318	1.0
LEBQ	SEGVBQ	0.7731	0.8167	0.3818	1.0
LEBQ	MEDVBQ	0.7731	0.8167	0.3625	1.0
UEBQ	SEGVBQ	0.7173	0.8167	0.0493	0.7402
UEBQ	MEDVBQ	0.7173	0.8167	0.05	0.7507
SEGVBQ	MEDVBQ	0.8167	0.8167	1.0	1.0

McNemar's test revealed significant differences in binary classification performance between UEBQ and several other metrics. Notably, UEBQ differed from mean VBQ (adjusted *p* = 0.0045), segmental VBQ (adjusted *p* = 0.0045), median VBQ (adjusted *p* = 0.0077), and LEBQ (adjusted *p* = 0.0016), with all comparisons remaining statistically significant after Bonferroni correction. This reflected consistent discordance in classification where UEBQ failed to identify cases detected by other predictors (Table 5).

**TABLE 5:** Mcnemar's test results.

Predictor1	Predictor2	b (1,0)	c (0,1)	Raw *p*‐value	Adjusted *p* (Bonferroni)
MEANVBQ	EBQ	10	2	0.0433	0.6496
MEANVBQ	LEBQ	3	5	0.7237	1.0
MEANVBQ	UEBQ	15	0	0.0003	0.0045
MEANVBQ	SEGVBQ	1	1	1.0	1.0
MEANVBQ	MEDVBQ	1	0	1.0	1.0
EBQ	LEBQ	0	10	0.0044	0.0664
EBQ	UEBQ	8	1	0.0455	0.6825
EBQ	SEGVBQ	1	9	0.0269	0.4029
EBQ	MEDVBQ	2	9	0.0704	1.0
LEBQ	UEBQ	17	0	0.0001	0.0016
LEBQ	SEGVBQ	4	2	0.6831	1.0
LEBQ	MEDVBQ	5	2	0.4497	1.0
UEBQ	SEGVBQ	0	15	0.0003	0.0045
UEBQ	MEDVBQ	0	14	0.0005	0.0077
SEGVBQ	MEDVBQ	1	0	1.0	1.0

### 3.6 Interobserver Reliability

The intraclass correlation coefficient (ICC) was calculated to assess the interrater reliability of the vertebral bone quality measurements. All variables demonstrated good to excellent reliability. The average measures ICC for UEBQ was 0.836 (95% CI: 0.720–0.904, *F* = 6.091, *p* < 0.001), for LEBQ was 0.858 (95% CI: 0.758–0.917, *F* = 7.049, *p* < 0.001), and for EBQ was 0.869 (95% CI: 0.776–0.923, *F* = 7.631, *p* < 0.001). The mean, segmental, and median VBQ demonstrated higher reliability with ICCs of 0.911 (95% CI: 0.848–0.948), 0.925 (95% CI: 0.871–0.956), and 0.902 (95% CI: 0.833–0.942), respectively (Table 6).

**TABLE 6:** Interobserver reliability.

Variable	Average measures ICC	*F* value	Sig	95% CI
UEBQ	0.836	6.091	< 0.001	0.720–0.904
LEBQ	0.858	7.049	< 0.001	0.758–0.917
EBQ	0.869	7.631	< 0.001	0.776–0.923
MEANVBQ	0.911	11.244	< 0.001	0.848–0.948
SEGVBQ	0.925	13.274	< 0.001	0.871–0.956
MEDVBQ	0.902	10.187	< 0.001	0.833–0.942

## 4 Discussion

### 4.1 Main Findings

This retrospective single‐center study demonstrates that higher MRI‐derived vertebral and endplate bone quality scores are strongly associated with cage subsidence following single‐level ACDF. VBQ metrics showed excellent discriminative performance and reproducibility, while lower endplate EBQ emerged as the most informative endplate‐based predictor. Differences between metrics became particularly apparent when applied as binary classifiers for risk stratification. Cage subsidence was observed in 46% of patients in our cohort, which may be considered an overrepresentation, considering studies report average rates of subsidence near 21%. This discrepancy may be attributed to variations in radiological definitions of subsidence, imaging modalities, and included patient populations [13]. Although definitions of subsidence vary across the literature, we adopted a ≥ 2 mm threshold to improve detection sensitivity in patients within our 6‐month follow‐up period. Only 16 patients in our cohort exhibited ≥ 3 mm of subsidence, limiting the statistical power of a sensitivity analysis using a higher cut‐off and increasing the risk of type II error. Importantly, while not the primary focus of our study, the trend toward greater loss of segmental and cervical lordosis among the subsidence cohort is important to note. Although these differences did not reach statistical significance, potentially due to the modest sample size, these findings may reflect subtle biomechanical consequences of cage subsidence in affected populations. The absence of any noticeable trend in loss of proximal junctional lordosis is likely attributed to its low incidence rate and the fact that only 36 patients were eligible for this parameter. Such findings underscore the recognition of radiologically detectable cage subsidence as not simply a benign imaging artifact, but as a possible hallmark of underlying structural alterations and potentially adverse outcomes.

### 4.2 Vertebral Versus Endplate Bone Quality and Risk of Subsidence

In our cohort, consistent with the literature, our study reaffirms poor bone quality as a risk factor and major contributor to cage subsidence. All VBQ and EBQ metrics were significantly higher among patients with subsidence, suggesting that fatty marrow infiltration may compromise overall vertebral and endplate integrity and predispose to implant migration. Overall, VBQ‐derived metrics seemed to perform similarly, with near‐identical AUCs, sensitivities, and specificities derived from ROC analysis. However, although they all demonstrated statistically significant correlations with cage subsidence in logistic regression, mean and median VBQ exhibited stronger associations compared to segmental VBQ, suggesting global marrow measurements may serve as a more robust predictor of structural failure than focal segmental measurements. Segmental VBQ, being confined to the operated levels, is more susceptible to variations in MRI signal intensity caused by local degenerative changes, disc pathology, or endplate irregularities, which may reduce its reliability as a consistent marker of bone quality compared to global VBQ assessments. Our findings also expand the clinical utility of EBQ scoring. Patients with higher EBQ scores, particularly at the lower endplate, had a greater likelihood of developing subsidence. There was more variability between endplate‐based metrics, with LEBQ scores demonstrating the strongest association with subsidence. These results support biomechanical theories suggesting that the inferior endplate is particularly vulnerable to mechanical compromise during axial loading in the context of fusion cages, thus reinforcing the clinical relevance of lower endplate bone quality as a more valuable predictive marker for subsidence risk [14].

### 4.3 Comparative Discrimination and Classification Performance

ROC analysis confirmed the superior discriminative capacity of VBQ‐based scores. Mean, median, and segmental VBQ scores all had AUCs above 0.81 and exhibited high sensitivity (92.3%) with reasonable specificity (73%–77%), highlighting their reliability in identifying high‐risk patients. In contrast, EBQ showed weaker diagnostic accuracy (AUC: 0.696), primarily due to low specificity. Interestingly, UEBQ demonstrated high specificity (90%), but poor sensitivity (0.538), limiting it as a standalone screening tool. LEBQ was found to possess superior discriminatory ability among the endplate‐based scores with an AUC of 0.773 while exhibiting high sensitivity (88.5%) and moderate specificity (63.3%). Overall, VBQ scores seemed to outperform EBQ scores in discriminative ability. Furthermore, while all metrics demonstrate a high degree of interobserver reliability, VBQ‐based measures all achieved ICC values above 0.9. In contrast, none of the endplate‐based measurements reached this threshold, suggesting that VBQ scores are more reproducible and offer greater consistency.

Pairwise comparison of predictive performance was conducted using both DeLong's test for differences in AUC and McNemar's test for differences in binary classification. DeLong's test revealed that mean and median VBQ scores significantly outperformed EBQ and UEBQ in terms of overall discriminative ability, with raw *p*‐values below 0.05 in several comparisons. For example, mean VBQ showed better ROC performance than EBQ (*p* = 0.0485) and UEBQ (*p* = 0.0425), while similar trends were observed for median VBQ compared to EBQ and UEBQ. Segmental VBQ also showed improved performance over EBQ (*p* = 0.0464). However, when adjusted for multiple comparisons using the Bonferroni correction, none of these differences remained statistically significant, with adjusted *p*‐values ranging from 0.637 to 0.754. This indicates that while trends favored the VBQ‐based metrics, the statistical evidence for superiority in AUC was not conclusive after correction for family‐wise error rate.

In contrast, McNemar's test demonstrated statistically significant differences in binary classification between UEBQ and multiple other predictors, and these findings remained significant after Bonferroni adjustment. UEBQ differed significantly from mean VBQ (adjusted *p* = 0.0045), median VBQ (adjusted *p* = 0.0077), segmental VBQ (adjusted *p* = 0.0045), and LEBQ (adjusted *p* = 0.0016), with each comparison showing high counts of discordant classification where the alternate metric correctly identified cases that UEBQ did not. For instance, the comparison between mean VBQ and UEBQ showed 15 patients classified as positive by mean VBQ but not by UEBQ, and none in the reverse direction. Similarly, the comparison between LEBQ and UEBQ showed 17 discordant cases in favor of LEBQ. These consistent patterns of directional discordance suggest that UEBQ frequently failed to classify cases that were correctly identified by other bone quality metrics. While comparisons between EBQ and other metrics also showed some discordance (e.g., EBQ vs. LEBQ: *p* = 0.0044), only comparisons involving UEBQ retained statistical significance after correction.

The apparent discrepancy between these two tests arises from their fundamentally different statistical aims. DeLong's test evaluates overall discriminative ability across the full range of possible decision thresholds, reflecting how well one metric separates outcomes in a continuous sense. In contrast, McNemar's test assesses how two metrics behave once a single optimal cutoff is applied, highlighting differences in case‐level classification rather than global performance. Thus, it is possible, as observed here, for two metrics to have similar AUCs yet differ significantly in their clinical utility when dichotomised for risk stratification. In this context, the significant McNemar results, both in raw and adjusted analyses, indicate that meaningful differences in classification behavior exist between the metrics, even when overall AUC discrimination does not differ statistically.

### 4.4 Comparison With Previous Studies

In the context of our findings, which suggest stronger overall performance of VBQ‐based metrics compared with EBQ for predicting subsidence after ACDF, it is notable that direct comparative studies remain limited. Several studies have explored the utility of both VBQ and EBQ scores in predicting postoperative outcomes following spinal fusion. Across all studies, the notion that both VBQ and EBQ are significantly associated with increased risk of subsidence remains consistent. However, there is disagreement regarding which metric provides superior diagnostic accuracy. Multiple studies have found that EBQ scores exhibit higher discriminatory capacity for cage subsidence and other outcomes such as adjacent segment disease when compared to VBQ [15, 16]. In contrast, other studies have reported that VBQ scores demonstrate greater overall predictive value, consistent with our findings [17]. It is important to note that these studies predominantly involve patients undergoing lumbar fusion procedures, thereby limiting the transferability of their findings in cervical contexts. As anatomical, biomechanical, and loading characteristics differ substantially between the lumbar and cervical spine, it is important to compare such results with caution. Moreover, the use of directly comparative statistical analysis such as McNemar's or DeLong's tests is not a commonly adopted method across such studies, and as a result, conclusions regarding superiority between these metrics lack robust statistical grounding.

### 4.5 Clinical Implications of Bone Quality Assessment

Although our study found significant correlation with only radiological subsidence and lacked patient‐reported outcomes, a growing body of evidence underscores its clinical relevance. Poor bone quality, as quantified by BMD, CT‐derived Hounsfield units, or MRI‐derived VBQ, has been linked to a range of adverse outcomes following spinal fusion, including adjacent junctional kyphosis and failure, reoperation, and hardware complications. Lower bone density measured by Hounsfield units has been correlated with distal junctional failure after multilevel fusions, while elevated VBQ scores independently predict proximal junctional kyphosis and failure in adult deformity surgery [18, 19]. Similarly, incorporation of VBQ into established risk scores enhances prediction of postoperative complications and reoperation rates, and higher VBQ values have been associated with an increased likelihood of revision surgery after lumbar fusion [20, 21]. Although some cervical studies report that subsidence alone may not directly affect long‐term outcomes if alignment is preserved, others show that subsidence is associated with segmental lordosis loss, which may contribute to pain, mechanical imbalance, and eventual clinical deterioration [22, 23]. Collectively, these findings indicate that radiologically detectable subsidence, and by extension the preoperative bone quality metrics that predict it, serve as meaningful surrogate markers of biomechanical compromise with downstream clinical consequences. Identifying high‐risk patients preoperatively using VBQ or EBQ scores could therefore guide implant selection, surgical technique, and perioperative management strategies to mitigate complications and improve long‐term functional outcomes.

### 4.6 Limitations and Future Perspectives

Despite the strengths of this study, several limitations must be acknowledged. First, the relatively small sample size, resulting from the strict inclusion and exclusion criteria, may limit the generalizability of our findings to broader clinical populations. This modest cohort size, particularly when subdivided into subsidence and non‐subsidence groups, also contributes to the wide confidence intervals observed in regression analyses and raises the possibility of type II error. Subtle associations such as differences in lordosis change or comorbidity effects may not have reached statistical significance due to limited power. Second, the retrospective design introduces inherent risks of selection and measurement bias, which may influence the observed associations. Third, subsidence was evaluated using follow‐up radiographs without dynamic positional standardization; although a ≥ 2 mm reduction in disc height was employed as a consistent threshold, subtle variations due to patient posture or radiographic technique cannot be entirely excluded. Fourth, while VBQ and EBQ serve as useful surrogates for vertebral bone quality by capturing fatty marrow infiltration, they remain indirect markers of bone mineral density and do not account for other structural contributors such as cortical thickness or trabecular architecture. Fifth, other covariates influencing cage subsidence such as BMI and DEXA‐derived bone mineral density were not consistently available in our cohort and were therefore not included in the analysis [24]. Finally, clinical and patient‐reported outcomes were not available, so the prognostic value of VBQ and EBQ in this study reflects radiological rather than functional endpoints. Taken together, these limitations suggest that while our findings provide important early insight into the predictive value of VBQ and EBQ scores for cage subsidence, further prospective, multicenter studies with larger cohorts, clinical outcomes, and standardized imaging protocols are warranted to validate and expand upon these results.

## 5 Conclusion

VBQ and EBQ scores are both valuable predictors of cage subsidence, with higher scores indicating poorer bone quality. VBQ metrics, especially global measures, outperformed EBQ in diagnostic accuracy, while LEBQ showed the strongest predictive value among endplate‐based scores. Significant classification differences, particularly with UEBQ, highlight the importance of metric selection. These findings support incorporating bone quality assessment into preoperative planning to identify high‐risk patients.

## Author Contributions

All authors had full access to the data in the study and take responsibility for the integrity of the data and the accuracy of the data analysis. Conceptualization: Omar Lubbad and Nektarios K. Mazarakis. Methodology: Omar Lubbad and Nektarios K. Mazarakis. Validation: Omar Lubbad and Nektarios K. Mazarakis. Investigation: Omar Lubbad, Wajeeh Ullah Mahmood, Rafiq Sheikhali, Abir Mamun, Laila Lubbad, and Akram Hagos. Formal analysis: Omar Lubbad. Writing – original draft: Omar Lubbad. Writing – review and editing: Omar Lubbad and Nektarios K. Mazarakis. Visualization: Omar Lubbad. Supervision: Giuseppe Morassi, Suzanne Murphy, and Nektarios K. Mazarakis. Project administration: Omar Lubbad, Suzanne Murphy, and Giuseppe Morassi. All authors listed meet the authorship criteria according to the latest guidelines of the International Committee of Medical Journal Editors and are in agreement with the manuscript.

## Funding

The authors have nothing to report.

## Disclosure

The authors have nothing to report.

## Ethics Statement

This study was registered with and approved by the Royal Sussex County Hospital trust committee and was conducted in accordance with the declaration of Helsinki. None of the authors have any financial or non‐financial competing interests to declare. There was no funding received for this study.

## Conflicts of Interest

The authors declare no conflicts of interest.

## Data Availability

The data that support the findings of this study are available on request from the corresponding author. The data are not publicly available due to privacy or ethical restrictions.
